# Bioelectronic microfluidic wound healing: a platform for investigating direct current stimulation of injured cell collectives†

**DOI:** 10.1039/d2lc01045c

**Published:** 2023-01-18

**Authors:** Sebastian Shaner, Anna Savelyeva, Anja Kvartuh, Nicole Jedrusik, Lukas Matter, José Leal, Maria Asplund

**Affiliations:** a Department of Microsystems Engineering, University of Freiburg Georges-Köhler-Allee 201 79110 Freiburg Germany; b Brainlinks-Braintools Center, Georges-Köhler-Allee 201 79110 Freiburg Germany sebastian.shaner@blbt.uni-freiburg.de; c Freiburg Institute for Advanced Studies (FRIAS), University of Freiburg Albertstr. 19 79104 Freiburg Germany; d Division of Nursing and Medical Technology, Luleå University of Technology 971 87 Luleå Sweden; e Department of Microtechnology and Nanoscience, Chalmers University of Technology Kemivägen 9 412 58 Gothenburg Sweden maria.asplund@chalmers.se

## Abstract

Upon cutaneous injury, the human body naturally forms an electric field (EF) that acts as a guidance cue for relevant cellular and tissue repair and reorganization. However, the direct current (DC) flow imparted by this EF can be impacted by a variety of diseases. This work delves into the impact of DC stimulation on both healthy and diabetic *in vitro* wound healing models of human keratinocytes, the most prevalent cell type of the skin. The culmination of non-metal electrode materials and prudent microfluidic design allowed us to create a compact bioelectronic platform to study the effects of different sustained (12 hours galvanostatic DC) EF configurations on wound closure dynamics. Specifically, we compared if electrotactically closing a wound's gap from one wound edge (*i.e.*, uni-directional EF) is as effective as compared to alternatingly polarizing both the wound's edges (*i.e.*, pseudo-converging EF) as both of these spatial stimulation strategies are fundamental to the eventual translational electrode design and strategy. We found that uni-directional electric guidance cues were superior in group keratinocyte healing dynamics by enhancing the wound closure rate nearly three-fold for both healthy and diabetic-like keratinocyte collectives, compared to their non-stimulated respective controls. The motility-inhibited and diabetic-like keratinocytes regained wound closure rates with uni-directional electrical stimulation (increase from 1.0 to 2.8% h^−1^) comparable to their healthy non-stimulated keratinocyte counterparts (3.5% h^−1^). Our results bring hope that electrical stimulation delivered in a controlled manner can be a viable pathway to accelerate wound repair, and also by providing a baseline for other researchers trying to find an optimal electrode blueprint for *in vivo* DC stimulation.

## Abstract 2

An on-chip bioelectronic platform for exploring precise direct current electric field stimulation of injured keratinocytes with salt-bridgeless electrodes.

## Graphical abstract

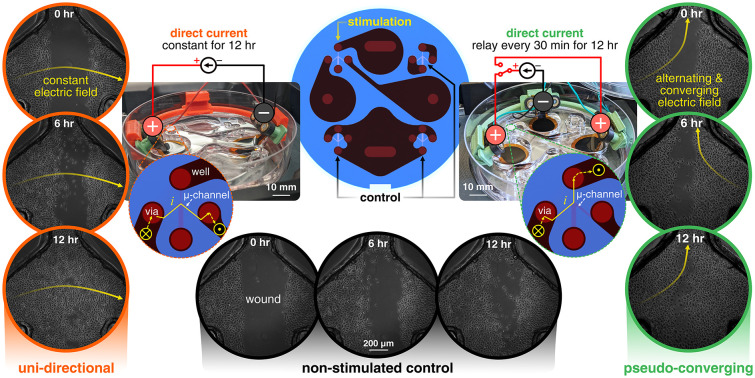

## 1 Introduction

For most of us, a wound is a minor nuisance, which heals itself without much conscious effort. However, for people with certain chronic diseases (*e.g.*, diabetes mellitus, peripheral vascular disease), compromised immune systems (*e.g.*, systemic lupus erythematosus), or even with common systemic factors such as poor nutrition and aging, acute wounds are more prone to become chronic. In fact, the high prevalence of chronic wounds constitutes an enormous socioeconomic burden (≈1 to 3% of the total healthcare spending in developed countries and growing as the median age of populations grows older),^1^ as well as suffering for the actual patients.^2,3^ Strategies to promote faster healing for these patient groups are therefore urgently needed. The healing process is often categorized into four sequential, yet overlapping phases: hemostasis, inflammation, growth, and maturation. There are many cell types involved in these concurrent phases (in general order of appearance): activated platelets, neutrophils, monocytes, macrophages, mast cells, dendritic cells, T cells, endothelial cells, pericytes, hematopoietic stem cells, fibroblasts, myofibroblasts, melanocytes, and keratinocytes.^4^ In the wound healing process these cell types are recruited, whether they are cells in close vicinity to the wound site or cells that have to traverse long distances *via* the circulatory system.

Chemical, mechanical, and electrical gradients all contribute to recruiting or guiding the aforementioned cells to the wound: processes called chemotaxis,^5^ haptotaxis/durotaxis,^6,7^ and electrotaxis/galvanotaxis,^8^ respectively. Notably, electrotaxis refers to the ability of cells to align their migration with electric fields (EFs). Neutrophils,^9^ monocytes,^10^ lymphocytes,^10^ macrophages,^11^ endothelial cells,^12^ fibroblasts,^13^ and keratinocytes^14^ have all been revealed to be electrotactic. Interestingly, wounds naturally form small EFs when the skin's epithelial layer is broken. This transepithelial potential (∼10 mV to 60 mV), which actively pumps sodium ions (Na^+^) basally inwards and chlorine ions (Cl^−^) apically outwards, is short-circuited after injury where positive current flows radially towards the wound center.^15^ There is a large inter-individual variability in the strength of this naturally-occurring (*i.e.*, endogenous) EF, which depends on the systemic nature of the patient (*e.g.*, age, disease). For example, it was shown that the lateral EF of 18–25 year-olds (107 mV mm^−1^ to 148 mV mm^−1^) is nearly 48% larger compared to that of 65–80 year-olds (56 mV mm^−1^ to 76 mV mm^−1^).^16^ Taken together with the fact that most skin cells exhibit electrotactic ability, the discovery of these endogenous EFs within wounds has led to the hypothesis that electrical cues are essential for migratory processes in wound healing.^17,18^ When it comes to exogenously supplying direct current EFs to wounds, there is evidence that undermines the significance of induced EF direction in *in vivo* wound healing. It has been indicated that applied ionic flow, regardless of EF direction, is a key driver in accelerating *in vivo* wound closure through up-regulation of mitogen-activated protein kinase (MAPK) signaling pathways and rapid reorganization of cellular components.^19–21^ Nevertheless, the hypothesis that the electrotactic-induced polarization of skin cells is paramount to properly distributed wound healing still remains an ever-evolving topic of interest, especially since earlier studies have not fully unraveled the merits of both EF magnitude and distribution on electrically-guided closure of physiological wounds.

Keratinocytes, the most prevalent cell type in the skin, are densely packed within any given lateral layer, and are also tightly arranged in vertical tiers (*i.e.*, stratified) where they become more differentiated the closer they get to the outermost, apical layer. In the skin, as well as in confluent cultured cell layers, keratinocytes migrate as a collective.^22^ Prior *in vitro* studies on electrotaxis of skin cells have typically focused on single cells, thus neglecting how the complex organization of cells in actual skin impacts the migratory behavior. However, collective cell migration is more indicative of *in vivo* cell dynamics for cell types like keratinocytes. On a group level, coordination within a cell collective starts with the protrusion of cells at the group's edge (*i.e.*, leader cells) and is propagated through cell-substrate forces (*i.e.*, traction of cell membrane-bound focal adhesions with the substrate)^23,24^ and cell–cell forces (*i.e.*, normal and shear forces *via* adherens junctions).^25,26^ The first demonstration of collective keratinocyte electrotaxis was shown by Zhao *et al.*, but they only explored EF directionality on single wound edges as compared to how endogenous converging EFs merge a perimeter of wound edges.^27^ Recently, there has been a resurgence in studying electrotaxis-mediated group migration with uni-directional^28–30^ and converging EFs.^31^ For instance, Zajdel *et al.* showed that two patterned monolayers of keratinocytes, with a 1.5 mm gap, can be influenced by an EF stimulus (*i.e.*, 200 mV mm^−1^) and close the gap between two epithelial sheets twice as fast as compared to non-stimulated controls.^31^ This work set an important precedent, and calls for further translational efforts to make EF-accelerated repair accessible to patients who are at risk of chronic wounds. A crucial step in this process would be to more closely replicate the process of wounding, where mechanical stress (*e.g.*, physical removal of cells) induces ATP and gap junction-mediated calcium waves across a monolayer of keratinocytes.^32^ Furthermore, as healthy skin typically heals well, it is paramount to analyse this effect in cultured disease models, which are associated with impaired wound healing and keratinocyte motility.

In this work, the aim was three-fold: (a) explore the influence of electrical guidance cues (EF distribution) on the rate of wound closure, (b) demonstrate how a non-metal DC stimulation electrode material can be stable and safe for cells even without typically needed salt bridges, (c) and establish a diabetic wound model to examine if electrical stimulation improves otherwise poor wound closure dynamics. In order to facilitate this, we developed a microfluidic version of the “classical” scratch wound assay, which allows us to explore the parameter space in which EF stimulation accelerates wound repair. Multiple fluidic concepts are analyzed to identify the layout that best mimics the standard scratch assay method, but with the superior experimental control provided by the microfluidic platform. Direct current (DC) compatible electrode materials are a key ingredient for EF stimulation *in vitro*, and will furthermore be essential for clinical translation.^33,34^ Here, we show that electrodes based on a combination of laser-induced graphene (LIG) and a PEDOT:PSS hydrogel, integrated within the platform, were capable of sustaining DC stimulation over hours. This is not only important for *in vitro* application, but likewise a prerequisite for subsequent clinical translation of the concept. Leveraging this wound-on-a-chip environment, we were able to explore the electrical wound healing concept, first for healthy cells and then using a culture-based model mimicking diabetic keratinocytes. Not only were we able to demonstrate that EF stimulation was effective for accelerating the wound healing for the healthy cells but also, importantly, we could restore the impaired mobility of diabetic-like cells as well.

## 2 Results

### 2.1 Microfluidic design for tailoring electric field distributions around wounds

An *in vivo* wound naturally generates an EF, which points radially towards the wound center.^35^ Electrically speaking, the wound center acts like a current sink (*i.e.*, cathode) surrounded by an ionic current source (*i.e.*, anode).^15^ When designing the microfluidic device, it is this principle that we mimic. An ideal setup would involve having an infinitesimally-small cathode at the wound center, such that all EF vectors guide electrotactic cells to the center. However, the limited ionic charge storage capacity of the electrode materials prevents such miniaturization. An alternative to miniaturizing the cathode, which can visually occlude the closing wound, is to explore how the layout of the microfluidic system can be tailored to allow EF stimulation to converge towards the wound center. Instead, our microfluidic design included a merging microfluidic network where the wounded cells will be centered and different combinations of electrode configurations around the wound can be explored.

**Fig. 1:**
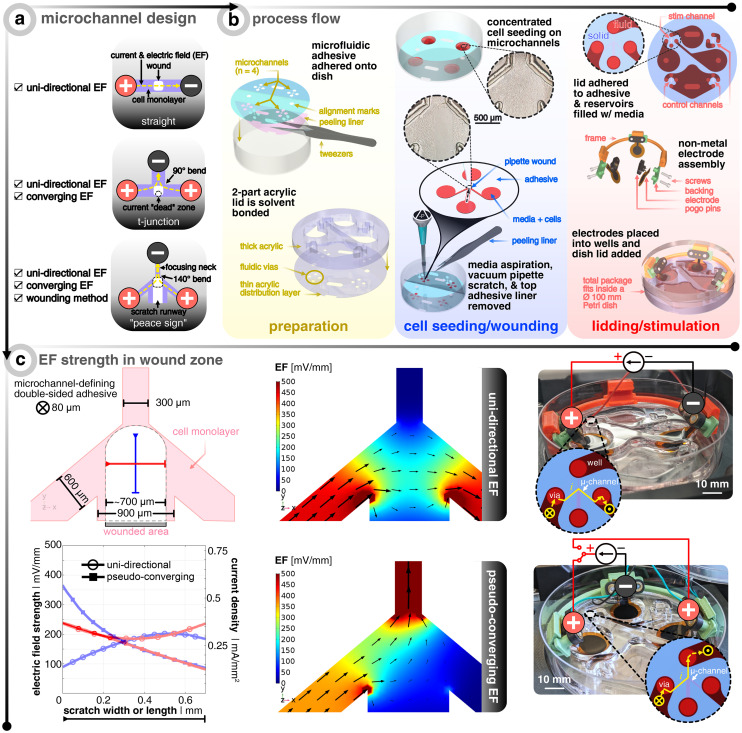
Microfluidic design to allow different electric field (EF) distributions around the wound. (a) Microchannel design options that were investigated. Red (+) signifies the anode(s) and black (−) denotes the cathode. Yellow dashed lines show the current and EF direction. Light purple shows the microchannel filled with a cell monolayer. White boxes show where the wound would be created. (b) Process flow of how the bioelectronic microfluidic platform is realized. For grouping of important steps, three panels are shown in the following sequential left-to-right order: yellow, blue, and red. In preparation (yellow), multiple microfluidic double-sided adhesive pieces are laser-structured, cleaned, and applied to standard Petri dishes. In parallel, two laser cut pieces of acrylic are solvent bonded to create microfluidic lids to be placed later. In cell seeding/wounding (blue), a high density suspension of keratinocytes is spotted over the open microchannels and allowed to seed before adding more medium and incubating overnight. Afterwards, a pipette tip is fitted to a vacuum aspirator to perform the wounding process. After wounding, the bulk medium is aspirated and the microfluidic adhesive's top protective liner is removed. In lidding/stimulation (red), the microfluidic lid is added to the newly-exposed adhesive to complete the device and medium is added to the microchannels and connecting reservoirs. Electrodes are assembled based on the desired EF strategy, and submerged into the reservoirs. The dish's lid is added and the complete package is placed into an incubated microscope. Note that each device has one channel for stimulation and three non-stimulated controls. (c) Finite element analysis of EF distribution within the microchannel for both electrode configurations where black arrows show the current direction and magnitude. The top illustration shows the dimensions of the microchannel and the typical profile of the wound. The orange and green lines show exemplary profiles to note the EF strength at the center of the wound in *x* and *y*. The bottom graph shows the aforementioned orange and green profiles for both electrode configurations. Note that an electrode input current of 25 and 20 μA is chosen so that center of the channel is about 200 mV mm^−1^ for the uni-directional and converging cases, respectively. The images on the right show the final realized devices, where the inset graphics show the current flow. The yellow X shows the current going from the open well into the closed microchannel and the yellow dot shows it coming out of the microchannel and into the well.

The core platform design question is how to design the microchannel network that houses the cells and wound (Fig. 1). While a standard straight channel design would lend itself nicely to applying an EF from one side of the wound (*i.e.*, uni-directional EF), one cannot provide a converging field. A “t-junction” design is another composition that could tackle both a uni-directional EF and a converging EF, depending on how the electrodes are placed and connected to the constant current source. However, in the converging case, the current will take the path of least resistance and turn the bend quickly into the cathode-containing channel, thus leaving a current and EF “dead zone” where the centrally-located wound resides (Fig. S1†). Upon computational investigation of different designs, we came up with a “peace sign” architecture, which mitigates the current “dead zone” by angling the anode-containing channels to reduce the current bending and by adding a fourth channel that enables a more practical physical removal of cells, which we call a “scratch runway” (Fig. S1†). The width of this fourth channel (900 μm) was chosen so that a p10 pipette tip (≃700 μm) could be used as the wounding tool, which is a typical way^36^ to create *in vitro* wounds. Then, this lower branch yields the option to have perfusive hydrostatic flow in order to ensure culture health throughout days-long experiments. In order to demonstrate this additional functionality, we used different colored dyes to validate that an effective converging flow can be accomplished without the use of any active component (*i.e.*, no perfusion pump or similar equipment needed) (Fig. S3†). However, for all functional tests, the perfusive flow option was not utilized as to decouple cell migration due to replenishing nutrients *versus* just the applied EF (12 hours long).

The next challenge is how to develop a scratch assay protocol inside a microfluidic channel where one can seed, grow, and then scratch a monolayer of cells all within an enclosed domain. Non-contact techniques of creating a scratch in a closed channel, such as UV exposure through a shadow mask or using a laser to ablate cells, fail to result in reproducible conditions as unsuccessful removal of debris has propagating negative effects on the leading edges of the wound. For this reason, we developed an approach that allows for cells to be seeded onto an adhesive-masked Petri dish where the cells adhere to the exposed area of the dish (Fig. 1). The protocol for making the electrotaxis wound healing device leverages common prototyping equipment (*i.e.*, CO_2_ laser) for both microfluidic adhesive structuring^37^ and electrode fabrication^38^ (see Methods section for more details). Using a double-sided adhesive with protective liners on both sides allows for removal of the bottom-side liner to adhere the exposed adhesive to the dish, all the while keeping on the top liner to protect the top adhesive from getting wet, thus acting as a sacrificial layer. Also adjacent to this step of device preparation, thick (8 mm) and thin (0.5 mm) acrylic sheets are laser cut to yield reservoirs/wells and fluidic *vias*, respectively. These two layers are solvent (*i.e.*, dichloromethane) bonded to act as the microfluidic lid and are set aside until the final assembly steps. Next comes cell seeding, overnight incubation, and subsequent injury of the cell monolayers in the traditional way (*i.e.*, mechanical removal *via* a pipette tip^36^) (Fig. S2†). We found that wedging a vacuum aspirator metal tip into a sterile plastic pipette tip allowed for more reproducible removal of cells with less cellular debris left in the wake of the scratch path. After wounding and aspirating of the bulk medium, the adhesive's top liner is removed so that the acrylic lid could be manually aligned and pressed onto the exposed adhesive to complete the final enclosure of the microchannels, which was then immediately filled with medium (Video S1† to see how the liner is peeled and the lid is attached). To be clear, the individual reservoirs are only fludically connected *via* the microchannels. A set of non-metal conducting hydrogel electrodes (*i.e.*, PEDOT:PSS hydrogel-coated laser-induced graphene) are assembled with custom 3D-printed pogo pin electrical connection adapters and holders. Note that the circles at the end of each branching microchannel act as the beginning of the open reservoirs, such that only the four branches are confined to a micron-size geometry in all dimensions. For further illustration of the fluidic architecture and reservoir/microchannel interface, see Fig. S1 and S3.†

Two configurations were analyzed in this study (Fig. 1c). The first will be referred to as “uni-directional EF” and corresponds to an EF across the wound (*i.e.* not a converging field). This strategy is the most straightforward to implement, as it requires only two electrodes, which are placed at either side of the wound (Fig. 1a). The electrotactic effect in this case will mainly apply to one side of the wound. If only electrotaxis-related forces would dominate the migratory behavior as seen in single cell keratinocyte cultures, then one would expect both wound edges to go with the EF direction and essentially move the same wound laterally.^14^ Nevertheless, the majority of studies that explore electrical wound healing *in vivo* exploit this type of setup, despite it not fully accounting for electrotaxis to act on both edges of the wound.^39,40^ The second configuration is here referred to as “pseudo-converging EF” since there is an anode on each side of the wound where a timed relay can switch between the two anodes to push the wound close from both sides in an alternating fashion. Consequently, in this scheme, the electrotactic effect will apply symmetrically to both sides of the wound. Additionally, the disconnected and polarized anode can passively recharge with ions from solution while the other anode is delivering charge.^34^

Using finite element analysis (FEA) of the three-dimensional microfluidic device, we identified the input current needed to achieve ≃200 mV mm^−1^ at the center of the wound, which has been shown to be an optimal EF strength for *in vitro* keratinocyte electrotaxis.^41–43^ There is a precedent that demonstrates how FEA simulation is accurate in predicting measured experimental EFs in microchannels.^44^ As validated by the FEA model, the geometric confinement around the monolayer of wounded cells provided by the microchannel allows for precise control of the field distribution (Fig. 1c). The current and normalized EF direction is indicated *via* the black arrows and the EF magnitude is depicted *via* the scaled color gradient and size of the black arrows. In order to visualize the EF magnitude along and across the wound, two profile lines are drawn (blue and red, respectively, in Fig. 1c). The plot shows the EF magnitude and corresponding microchannel current density along these lines for a given electrode input current (25 μA and 20 μA for the uni-directional and pseudo-converging cases, respectively). The hollow circles show that the uni-directional case yields a more uniform EF distribution across (*i.e.*, *x*-direction, red) and along (*i.e. y*-direction, blue) the wound compared to the pseudo-converging case. The center of the wound is defined to be where both lines intersect (for a comparison to non-angled branched designs, see Fig. S1†). The input currents were selected to match a value of 200 mV mm^−1^ at this intersection point.

### 2.2 Minimal pH shifts and joule heating during direct current stimulation

**Fig. 2:**
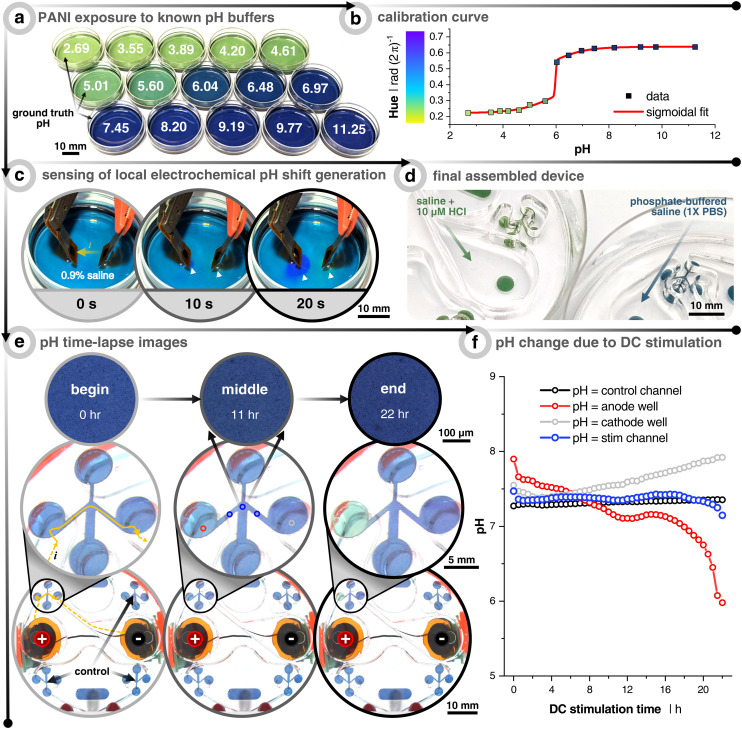
Measuring pH shifts due to direct current (DC) stimulation. (a) Polyaniline (PANI) coated dishes subjected to solutions already measured using a benchtop pH meter. (b) Calibration curve relating the hue of the PANI-coated dishes to the pH. The curve fitting was done with a “bi-dose response” sigmoidal fit. (c) Demonstration of how the PANI-coated substrate can sense local electrochemically-induced changes in the pH. Cathode is on the left and anode on the right. Electrodes used are the same materials, but smaller (3 mm diameter), as the electrodes used throughout the paper. Solution is unbuffered 0.9% NaCl and current is constant at 0.40 mA. White arrows point to local pH changes. (d) Example of the final assembly of PANI-coated scratch assay devices when filled with hydrochloric acid (HCl) spiked saline (pH = 5.4) and phosphate-buffered saline (pH = 7.4). (e) Time-lapse example images of 1× phosphate-buffered saline (PBS) filled device with PANI-coated microchannels. The stimulation protocol is a uni-directional EF configuration and stimulated for 22 h at 25 μA (see Fig. 1). Yellow arrows show the current path from the reservoir down into and across the microchannel. Note that the images on the bottom two rows were taken with a wide-field camera, whereas the those on the top row were taken with a 5× objective on an incubated (37 °C & 5% CO_2_) microscope. All quantitative data were plotted using the 5× images in order to minimize any impact from ambient light fluctuations. (f) Quantitative output of pH changes as a function of DC stimulation time. Colors correspond to the imaging locations shown in (e) of the center image. The black trace represents the non-stimulated control microchannel. The blue trace represents the average of 3 locations (before, center, and after wound zone) of the stimulated microchannel. The red and gray traces are taken at the interface between the reservoir and microchannel entry/exit, which show the anode and cathode reservoirs, respectively.

For reproducibly generating electrotactic behavior, it is fundamental that electrotactic effects are decoupled from other possible interferences. For instance, faradaic reactions at the electrode–electrolyte interface can induce redox reactions, which lead to a lowering of pH at the anode (*i.e.*, more H^+^) and a rise of pH at the cathode (*i.e.*, less H^+^). Since previous studies have explicitly pointed to pH as a determinant factor of electrotaxis^45^ and since the confined volume inside the microfluidic compartment causes the system to be more sensitive to such variances, we explicitly validated the pH stability within the microfluidic system under DC stimulation. In order to monitor the pH, the microchannel floor is coated with the colorimetric pH-sensitive polymer polyaniline (*i.e.*, PANI) (Fig. 2, Video S1†). The color changes with the oxidation state of the conjugated polymer. At low pH (≃3 to 5), PANI is in its emeraldine salt oxidation state and gives a gradient color from yellow-green to dark green as the pH increases. At a more neutral pH (≃6 to 8), PANI gradually changes to its emeraldine base oxidation state and begins to transition from dark green to green-blue to blue. Finally, at higher pH (≃9 to 12), PANI progresses to a fully oxidized state called pernigraniline, where the color goes from blue to dark blue to dark purple.^46^

The purpose of the PANI coating is to identify at which cutoff time the DC stimulation will induce notable pH shifts in the microchannel for the given input currents. First, PANI's pH-sensitivity was tested by coating a thin layer onto small Petri dishes and filling them with 15 known pH buffers to create a calibration curve relationship between the pH and the consequent PANI color (*i.e.*, hue) (Fig. 2a and b). The sensitivity to indicate pH change during 20 s of DC stimulation was first verified using a non-buffered saline solution (0.9%) with relatively high current density (5.7 mA cm^−2^), which is about 250× greater than what was planned for the microchannels, in order to rapidly see pH changes below the electrode pair. As soon as 10 s into stimulation was reached, the area under the cathode and anode began to turn more basic (purple) and acidic (green), respectively (Fig. 2c). This demonstrated PANI's ability to display rapid visual pH dynamics as a function of electrochemical faradaic by-products before moving into the device architecture.

A phosphate-buffered saline (PBS) solution of pH 7.4 is used as the testing electrolyte, which also has a similar osmolarity and conductivity to the keratinocyte medium. PANI is coated in the microchannel analogous to the way cells are seeded in the device (Fig. 1b, step 3 and 2d). We here focused on the uni-directional EF case, which represents the highest current injected into the system, and therefore can be expected to correlate with stronger potential pH shifts. Also, the pseudo-converging EF does not induce an acidic pH swing of the same degree due to minimizing the injected faradaic current by way of using two anodes that rely on relay-switching and passive ion recharging (Fig. S4†). Furthermore, the spatiotemporal color/pH stability is verified using the non-stimulated control channel (Fig. 2f – black). Imaging is performed in two ways. The first experiment was carried out with a transmission light microscope fitted with a 5× objective to control light intensity and minimize ambient fluctuations (Fig. 2e, top row). The second was performed with a wide angle camera lens to concurrently capture a global view of all control and stimulation channels (Fig. 2e, middle and bottom rows). As expected, PANI at the transition between the anode reservoir and microchannel (*i.e.*, left circle and channel) turns more acidic and *vice versa* for the cathode well (Video S2†). From this, we conclude that at least 12 h of DC stimulation is possible without inducing significant pH shifts in the microchannel's wound zone for this combination of input current, electrode size, stimulation configuration, pH-buffering capacity, and microfluidic design. We would like here to emphasize that no cross-flow was used to dilute potential electrochemical by-products, and also no salt-bridges nor any other supporting systems are utilized, which otherwise are essential components when using other metal-based electrode systems.

Another possible interference of applying DC across a fluidic resistor (*i.e.*, microchannel) is that joule heating effects could increase the metabolism of the cells and allow them to migrate faster. In order to account for this, it is important to have an idea of the amount of joule heating as a function of DC stimulation time. Thermocouples are susceptible to the effects of electromagnetic fields, particularly induced voltages from the EF, and are typically physically much larger than the microchannels employed in this work. Therefore, we opted to focus on a FEA simulation-based approach. Specifically, joule heating effects within the electrolyte were computed using the multiphysics coupling of electromagnetic time-independent equations (current conservation based on Ohm's law and scalar electric potential) and heat transfer time-dependent equations (energy conversation using Fourier's law). Both uni-directional and converging cases are explored within a 12 h stimulation cutoff. The model involved simplifying the geometry to just the stimulation microchannel network, connected reservoirs, and surrounding plastic substrate and lid, as well as the electrodes sitting on top of the reservoirs (Fig. S5†). Even after an energy transfer of 2.74 J (25 μA, 12 h, 101.6 kΩ) and 1.87 J (20 μA, 12 h, 108.3 kΩ) for the uni-directional and converging EF cases, respectively, the temperature in the wound zone only rises by less than 0.1 °C. Since more current is forced through the smallest width microchannel branch in the pseudo-converging EF case, the electrical resistance was higher leading to higher joule heating (the maximum temperature increase was 0.03 °C and 0.11 °C after 12 hours of DC stimulation for the uni-directional and pseudo-converging cases, respectively). Using the combined experimental and computational approach to account for pH shifts and joule heating due to DC stimulation, we could conclude that the cells are safe from electrochemically-induced faradaic reactions and heat-induced apoptosis during the 12 h stimulation of cells in our platform at these currents.

### 2.3 DC stimulation expedites wound closure of keratinocytes

Preliminary electrotaxis studies of single cell keratinocytes are in consensus that there is cathodic directionality of migration, but there are mixed reports on small or significant increases of migration speed compared to non-stimulated controls.^42,43,47^ In the body, however, keratinocytes are organized as packed layers, and only a few studies have attempted to study the more skin-relevant situation of electrotaxis in confluent cell layers. For *in vitro* electrotaxis of keratinocyte monolayers, evidence has consistently shown an increase in motility compared to non-stimulated controls.^27,41^ Recently, it was shown that monolayers of keratinocytes have both cathodic migration directionality and increased migratory speed (∼3×) when subjected to an external direct current EF (200 mV mm^−1^).^41^ Globally, collective cells move like an elastic material with a constant tug-of-war between the cells at the advancing edge (*i.e.*, leader cells) and the conglomerate of cells behind them (*i.e.*, follower cells), where there is an interplay of forces that act on the individual and collective group levels.^26^ This is where collective cell migration and individual cell migration differ as the latter is not directly influenced by their neighbors, thus highlighting the importance of studying the more wound-realistic crowd migration of damaged epithelial sheets whose leader cells are steering the way.

**Fig. 3:**
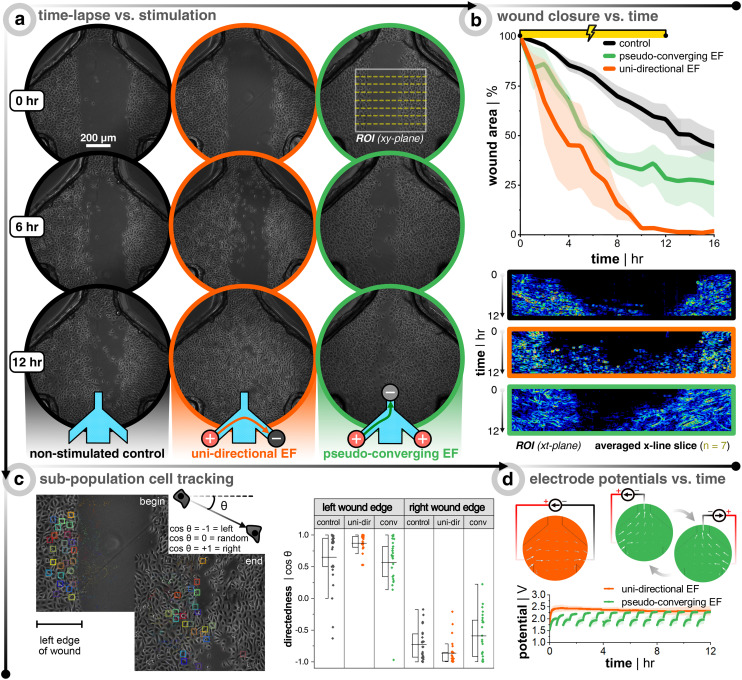
Bioelectronic wound healing assay of healthy keratinocytes demonstrates faster wound closure with stimulation. (a) Time-lapse images during 12 h DC stimulation for non-stimulated control (black, left panel), uni-directional EF (orange, middle panel), and pseudo-converging EF (green, right panel). (b) Plots of the wound closure over time, where the wound area is normalized to the first image (*n* = 3 for all conditions). Below the plots are the corresponding kymographs of the wound region of interest (ROI). Each ROI has seven line slices in the *x*-direction across the wound and these seven lines are averaged and stacked for each time point in the kymograph (image taken every 10 min for 12 h of stimulation yields 72 rows of averaged pixels). The kymograph color scale corresponds to the phase-contrast image intensity. (c) Cell tracking of a sub-population of cells from both wound edges. The directedness of the cell's path is determined by noting the *xy*-location at each frame and calculating the cosine of the displacement angle. A value of −1 shows directed migration to the left, +1 shows migration to the right, and 0 would show non-directed migration. (d) Example profiles of potential *versus* time for both electrode configurations. Note that for the converging case, an extra anode is connected (compared to the uni-directional case) and a relay switches the anode every 30 min to push cells from both sides of the wound, as well as passively recharging the unconnected anode with ions from the medium.

Healthy keratinocytes are seeded, grown to full confluency, wounded, and DC stimulated according to Fig. 1. The stimulation protocol was either 12 h of a uni-directional or pseudo-converging EF, and each stimulation replicate had multiple internal non-stimulation controls. Remarkably, DC stimulation resulted in faster wound closure in all cases (Fig. 3a and b). At the end of stimulation, the wounds subjected to a uni-directional EF (*n* = 3, in orange) were ≈100% closed, those with a converging EF (*n* = 3, in green) were ≈72% closed, and the controls (*n* = 9, in black) ≈42% closed. For the uni-directional EF case, the effect was even stronger and the full wound closure was seen even at 10 h when the controls were only ≈36% closed, which is nearly a 3× increase in closure rate. Kymographs are provided for each case in order to show how the wound closes over time for all 72 frames (Fig. 3b, bottom row). Each row of pixels in the kymograph corresponds to a specific time point where seven lines within the wound region of interest are averaged, plotted, and color-mapped in correlation to the phase-contrast image's intensity. Cell tracking also confirms that a uni-directional EF promotes more directedness across the wound and a larger displacement compared to the non-stimulated control (Fig. 3c). If the wound closure was purely following the logic of electrotactic behaviour in single cells, one would expect that the closure speed would be faster for the converging EFs, where both edges of the wound experience a field that should drive them towards the wound center. The faster closure speeds were instead seen for the uni-directional EF, which is rationalized in the discussion. Cell tracking showed that the anode switching in the pseudo-converging EF case did not provide a boost in lateral directed migration, but rather more of a vertical directed displacement as compared to the non-stimulated control (Fig. S6†). In order to confirm the direct influence of the current and EF direction on group migration, a supplementary pseudo-converging EF stimulation experiment where the current polarity was toggled between ±20 μA was performed to show migration direction reversal (Video S5†). Note that the pseudo-converging EF stimulation scheme in Fig. 3 involved a relay-based switching of the left and right anodes every 30 min to allow for cyclic passive recharging of the non-connected and depleted anode with cations from solution, which can be seen in the rapid rising potential at the beginning of each discharge that is predominately due to capacitive current (Fig. 3d). This scheme allowed the electrodes to operate at a lower potential, which would be beneficial to reduce electrochemical side-effects. There was a subtle increase of the potential over time, which is likely due to the sudden switching of anodes adding stress on the cathode. Owing to the superior wound closure performance of the uni-directional EF scheme, it was opted to be the focal stimulation case for the diabetic model of keratinocytes.

### 2.4 DC stimulation promotes recovery of inhibited keratinocyte wound closure motility

In order to explore the hypothesis that EF stimulation not only accelerates wound closure for healthy cells, but also is of relevance for patients with impaired wound healing, it is compelling to establish a protocol to mimic the less motile wound closure phenotype of diabetic wounds. Once established, this could be translated to testing inhibited cells under a direct current EF to see if it helps recover the lost motility. Two approaches to model diabetes are tackled in this paper. The first is to subject the seeded keratinocytes to a hyperglycemic environment (*i.e.*, high glucose concentration), which restrains migration speed *via* sequential suppression of the p-Stat-1 pathway and the α2β1-integrin-mediated MMP-1 pathway.^48^ The other approach is to inhibit the p38 mitogen-activated protein kinase (MAPK) pathway. This pathway is directly involved in the transition of keratinocytes from cells which are destined to terminally differentiate into the outermost skin layer (*i.e.*, stratum corneum), and helps transform them into highly migratory cells upon wounding, which is part of the regeneratory process.^49,50^ The p38/MAPK pathway has been shown to be activated during the epithelial regeneration process.^51,52^ This same pathway has been shown to be down-regulated in high glucose environments, which is a common phenotype of diabetic wounds.^53,54^ Thus, if this pathway can be restored (up-regulated), then it might be possible to restore the cell migration *via* an autophagy-dependent manner.^54^ Jiang *et al.* showed that down-regulation of CD9, a gene encoding protein involved in cell motility, promotes keratinocyte migration. Also, p38/MAPK inhibition increases CD9 expression, thus suppressing migration.^52^ Building off that knowledge, it was also shown that keratinocytes subjected to direct current EFs (200 mV mm^−1^) will have their CD9 expression down-regulated *via* the 5′ adenosine monophosphate-activated protein kinase (AMPK) pathway.^55^ The sum of these factors led us to believe that if keratinocytes are slowed *via* an inhibited p38/MAPK pathway (increased CD9 expression), then direct current EFs could down-regulate CD9 and override the migration inhibition *via* the alternative AMPK pathway.

**Fig. 4:**
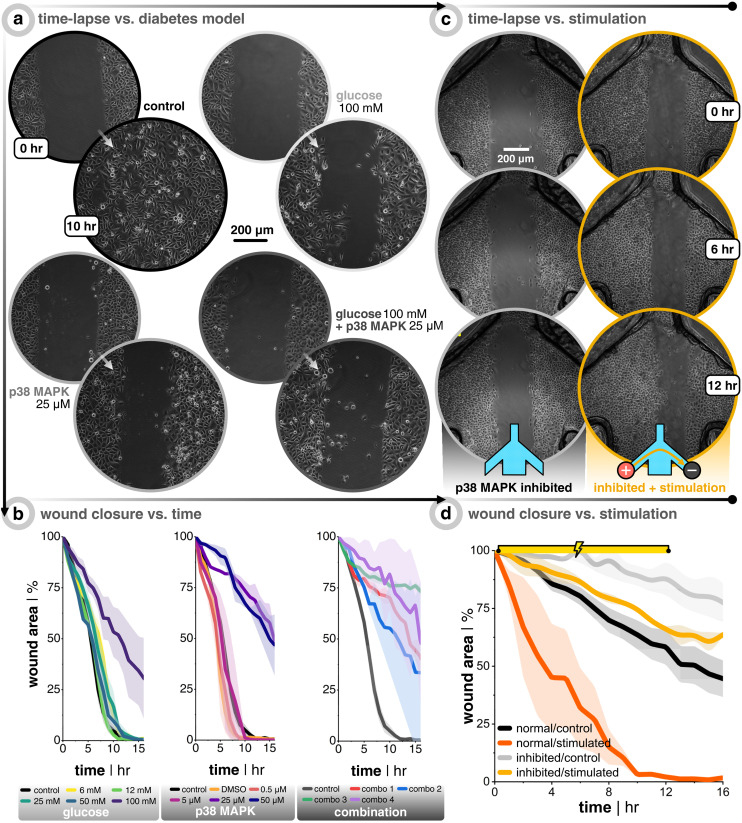
Inhibitory treatments of keratinocytes slow down migration and DC stimulation helps recover this lost motility. (a) Keratinocytes seeded on a 12-well plate are subjected to normal growth medium (control), medium with d(+)-glucose, medium with p38/MAPK inhibitor, or a combination of both. After treatment, a scratch assay is performed and time-lapse images are taken. (b) Plots of the wound closure over time, where the wound area is normalized to the first image (*n* = 3 for all conditions). Note that the p38/MAPK inhibitor stock solution is diluted with dimethyl sulfoxide (DMSO). Therefore, a control group was added with the same final concentration of DMSO that was used in all inhibitor cases in order to account for DMSO's effect on wound closure. (c) Time-lapse images during 12 hour DC stimulation for non-stimulated control (in grey) and uni-directional EF (in gold). (d) Plots of the wound closure over time, where the wound area is normalized to the first image (*n* = 3 for all conditions). The orange and black traces are a carry-over from Fig. 3 in order to facilitate comparison with healthy keratinocytes.

After seeding keratinocytes so that they were fully confluent the next day, they were subjected to either keratinocyte growth medium spiked with different concentrations of d(+)-glucose (6 mM to 100 mM), medium spiked with p38/MAPK inhibitor (0.5 μM to 50 μM), or a combination of both (Fig. 4). The cells were kept in the glucose environment overnight before wounding and imaging, while the p38/MAPK inhibited cells were subjected for 3 h. Importantly, all inhibitor treatments tested did not affect cell viability even after 24 h of treatment (Fig. S7†). Both treatments on their own were successful at slowing down wound closure, starting at 100 mM for glucose and 25 μM for p38/MAPK inhibitor. The wound closure rate was more than halved compared to the untreated controls (Fig. 4b, black trace, Video S3†). The combinations of both treatments (the amalgam of 50 or 100 mM glucose and 25 or 50 μM inhibitor) were also successful at reducing migration speed, but were much less consistent amongst replicates compared to the single treatments (Video S4†). All things considered, it was decided that the effectiveness and reproducibility of p38/MAPK inhibitor (25 μM) were the best conditions moving forward since it decouples possible compounding issues of dual treatments, in addition to targeting a specific pathway that is potentially more directly linked to electrotaxis.

Importantly, the positive effect of EF stimulation on wound closure was demonstrated here to hold true also for p38/MAPK-inhibited keratinocytes. It was clear that direct current EF guidance cues help close the wound faster than non-stimulated controls (Fig. 4c). After 12 h of uni-directional EF stimulation, inhibited cells (*n* = 3, in gold) were ≈34% closed compared to only ≈12% closed for non-stimulated controls (*n* = 3, in grey). To put this ∼3× increase in closure speed into perspective, DC stimulation aided the inhibited cells to nearly recover to the wound closure speed as the once healthy keratinocytes (Fig. 4d, gold *vs.* black traces, Video S6†). Our findings support the idea that electrical field guidance can act to support faster wound closure and, in particular, the potential relevance for addressing impaired wound healing associated with diabetes.

## 3 Discussion

The harmonization of microfluidic design, electrode material choice, and assembly protocol that were shown in this work provides a new platform for exploring the effects of electric fields on wounded cells of choice. The platform is based on readily-available materials and commonly-used prototyping equipment, and could easily be manufactured and customized by others according to our protocols. Below, we discuss three major topics that were leveraged in this work, their key parameters, and the future outlook: DC electrode materials, guidance cues, and disease models.

A major hurdled obstacle that we report here is the application of electrodes that do not require salt bridges, which allows for easier translation to 3D models.^33^ The use of recently developed non-metal, supercapacitive hydrogel electrodes facilitates not only a compact design in this 2D assay work, but also ease of translation to more 3D architectures, and importantly, it is an enabling technology for translation into a clinically useful device.^38^ However, it is of critical importance to focus on the electrode's charge storage limitations and the stimulation protocol's total charge to be delivered. The pseudocapacitor hydrogel electrode used here has a relatively high charge storage capacity (CSC ≃ 40 mC cm^−2^ to 50 mC cm^−2^).^38^ This CSC is crucial for determining over how long the current can be delivered capacitively (*t* = *q*/*i*, where *q* is the charge stored in the electrode and *i* is the input current) before it shifts to a predominately faradaic current, which is reliant on pH-shifting electrochemical reactions. This is why it is imperative to also account for the amount of pH buffering agent (molarity). On the one hand, the availability of nearby buffering agents in *ex vivo* or *in vivo* constructs might not be as abundant as in carefully engineered *in vitro* systems; however on the other hand, perfusion in functional tissue can help supply reinforcing buffering agents. Additionally, we demonstrate that over-polarized electrodes after stimulation are able to recharge with ions from the surrounding biological electrolyte and that switching between two anodes mitigated faradaic-induced pH shifts at the anodes (Fig. S4e†). This opens up possibilities to employ multi-electrode arrayed anodes and cathodes that have sub-groups that actively discharge (*i.e.*, unipolar, constant current), while other sub-groups passively recharge (*i.e.*, disconnected), and then cyclically interchange between these two sub-groups. It should be noted here that metal electrodes typically do not possess this ability to recharge, as their DC charge injection involves corrosion, a reaction that cannot easily be reversed. In addition, corrosion typically elutes toxic metal ions into tissues. Meanwhile, the metal-free electrodes used here work with ions available in abundance in the biological electrolyte.

Right now, the fields of bioelectronics and wound healing are just ‘scratching’ the surface of how best to use guidance cues to control cell collectives. This work unveiled the surprising result that constantly pushing a wound to close from one side *via* EF guidance cues was more effective than alternatingly directing the wound to close from both sides. At its face value, this shows electrotaxis, particularly the EF direction, is not the only main driver in accelerated *in vitro* wound closure *via* electrical stimulation. Since the wound edge that needs to travel against the EF direction is still able to close the gap, albeit slower, this suggests either kenotaxis,^24^ free edge motility due to activation of epidermal growth factor receptor (EGFR),^56,57^ or up-regulation of MAPK pathways due to exogenous ionic flow^19^ are supportingly important in collective cell wound healing in the presence of electrical stimulation. As for why a pseudo-converging EF provided slower wound closure compared to a uni-directional EF, we hypothesize that this is due to time-varying shifts in the polarization direction of the leader cells, which could have compounding negative traction effects each time the anode is switched. This compounding effect could also explain why the initial slopes of closure rates were similar for both stimulation schemes, but ultimately slows down for the pseudo-converging EF case. These uni-directional EF results can be leveraged when designing future electrical wound dressings since a truly converging EF design would require the problematic task of placing a cathode within the wound's exudate. Furthermore, with an extensive variety of wound morphologies, the centrally-placed cathode becomes more difficult to standardize, whereas this burden would be less so with the simplicity of fixing an electrode on opposite sides of the wound. Here, additional electrode montages could be employed to focus on wound closure from different sides or edges. With that being said, there is still a need to dive further into how guidance cues impact group migration. Recently, Shim *et al.* demonstrated that increasing levels of calcium proportionally increases cadherin-mediated cell–cell adhesion strength in between adjacent keratinocytes, reduces the average cell migration speed, and dampens the effect external direct current EFs has on directionality, which goes to show there is an interplay of cell–cell forces, cell–substrate forces, and external guidance cues in effective collective migration control.^58^ In conjunction with bioelectric cues, mixtures of mechanical cues (*e.g.*, extracellular matrix coatings) and chemical cues (*e.g.*, passive or active flowing of pro- or anti-migratory soluble factors) are compelling to investigate and straightforward to implement in a platform such as the one presented here. Also, it is feasible to leverage microfluidic laminar flow to cleverly focus the flow^59^ of compounds of interest in a spatial manner (also possible here in Fig. S3†). These external cue-mediated 2D sheet migration insights may unveil new mechanisms, and are of importance for translating findings from single cells to more complex and tissue-relevant architectures.

Modeling a disease *in vitro* has its limitations, but offers opportunities to study individualistic cause and effects. Diabetes *in vitro* models have been thoroughly explored in the aspects of neuropathy,^60^ pregnancy,^61^ and wound healing.^54^ Hyperglycemic *in vitro* studies of wound healing rates seem to depend on the epidermal growth factor (EGF) concentration in glucose-spiked media, which suggests that solely using glucose concentration as the independent variable is not targeted enough.^62^ However, targeting downstream effected pathways of hyperglycemia, like the p38/MAPK pathway, offers a more directed approach to induce a diabetic-like state in the most wound-prevalent cells, keratinocytes.^53,54^ Prior studies have generated evidence that other pathways also play an influential role in diabetic wounds, including the diacylglycerol pathway, hexosamine pathway, protein kinase C pathway, and polyol pathway.^63^ This illustrates that there is more room to explore the impact of multiple guidance cues on a variety of relevant diabetic wound pathways at the foundational level. The treatment of healthy keratinocytes was used in this study in order to facilitate direct comparison using the same cell line, culture medium, and seeding methodology. However, it has its limitations in fully encompassing diabetic keratinocyte behavior. Using the presented bioelectronic platform, investigating keratinocytes from diabetic patients and even inducing type 2 diabetes hallmarks in healthy keratinocytes *via* exposure to diabetic fibroblasts are both fruitful alternatives worthy of future investigation.^64^ In addition to healthy and diabetic keratinocyte collectives, co-culture models are of interest as they introduce more intercellular crosstalk and potential collision dynamics that were not explored in this work, but nonetheless, could be investigated using this platform. The next logical step of 3D human diabetic skin equivalents^64,65^ could also be potentially explored by substituting the cell seeding step for inserting pre-formed skin constructs into the open microchannel before lidding.

Aside from diabetes, more ambiguous diseases like systemic lupus erythematosus (SLE) also have affected wound healing. Diseases like SLE stand to benefit from more fundamental research on responsible pathways that could potentially be overridden by electrical stimulation in order to improve their typically poor healing of wounds. There is also evidence that transcutaneous electrical stimulation promotes healing of intact skin for such ailments like pressure ulcers of paraplegic individuals.^66^ However, for all these aforementioned diseases, there needs to be more exploration into the mechanistic effects of electrical stimulation and more encompassing dose–response investigations from the collective cell group scale all the way up to the organ level. Electrical stimulation on the single-cell level to multicellular 3D constructs all require careful consideration of how and where the current and EF pass through target regions of interest. The microfluidic regime helps channel and direct EFs in a predeterministic fashion during the design phase, which unlocks robust stimulation platforms to study phenomena beyond just wound healing.

## 4 Conclusion

Compared to the canonical wound healing model, this bioelectronic microfluidic platform enables new grounds to directly investigate the role of precise delivery of EF magnitudes with different options for EF directionality in wound closure. This level of EF controllability could not be achieved by simply submerging electrodes in the conventional wound healing platform of seeded well-plates. While this demonstration used seeded keratinocyte monolayers, it could also be utilized with tissue models by replacing the seeding, growth, and wounding steps with the placement of tissue in the microchannel before lidding. Furthermore, this platform allowed us to lay the foundation for a future wound healing concept based on electrical stimulation from supercapacitive non-metal electrodes. We demonstrated the working principle of this concept using culture models of skin wounds, and showed that EF guidance cues can increase the wound closure speed up to 3×, in comparison to non-stimulated controls. We furthermore showed that effective wound closing stimulation relies on a carefully controlled environment, dosage, and directionality of the electric field. Under the conditions that these factors can also be accounted for in real skin wounds, we are convinced that electrical stimulation could contribute as an additional guidance cue in regenerating tissue and thereby promote faster re-epithelialization.

## 5 Methods

All chemicals were purchased from Sigma Aldrich, unless otherwise noted.

### 5.1 Finite element analysis of electric field distribution and joule heating

COMSOL Multiphysics® software (version 5.3) was used to simulate both EF distribution and joule heating, using the Electric Currents and Heat Transfer modules, respectively. SolidWorks (version 2021) was used to design the three-dimensional models used for COMSOL. For the EF distribution, electrodes sat on top of the reservoirs and were modeled to have the electrical conductivity of PEDOT:PSS hydrogels (*σ* = 2000 S m^−1^).^67^ The medium was modeled after 1× (*i.e.*, 10 mM) phosphate-buffered saline (PBS, *σ* = 1.54 S m^−1^). Only the form factor of the medium and the electrodes were modeled. The input current density (placed at the face of the anode(s)) was sweeped in order to identify at which current the desired EF strength would be reached at the center of the channel. The cathode was set to a potential of 0 V. For joule heating, the same electrical conditions were implemented, but changed to a time-dependent solver. This model also included modeling fully medium-filled reservoirs and the plastic substrate and lid. The following values were used for heat transfer-related material properties (values are from COMSOL's material database unless otherwise cited): PBS (*k* = 2 W m^−1^ K^−1^, *ρ* = 1000 kg m^−3^, *C*_p_ = 4 J kg^−1^ K^−1^);^68^ acrylic (*k* = 0.19 W m^−1^ K^−1^, *ρ* = 1190 kg m^−3^, *C*_p_ = 1420 J kg^−1^ K^−1^, *σ* = 1 × 10^−14^ S m^−1^); PEDOT:PSS (*k* = 0.348 W m^−1^ K^−1^,^69^*ρ* = 1060 kg m^−3^,^70^*C*_p_ = 1415 J kg^−1^ K^−1^).^71^ The initial conditions include a convective heat flux of external temperature of 310.15 K with a heat transfer coefficient of 5 W m^−2^ K^−1^, a diffusive surface of all plastic components with a surface emissivity of 0.95 and an ambient temperature of 310.15 K. The fluid was modeled to have zero velocity and a pressure of 1 atm.

### 5.2 Microfluidic fabrication

All components of the microfluidic device, besides the sterile polystyrene dish, were fabricated with a 30 W carbon dioxide (CO_2_) laser (Universal Laser Systems, VLS 2.30). Specifically, 7.5 W at 70 mm s^−1^ was used for a kiss-cut and 24 W at 70 mm s^−1^ was used for a through all-cut for the acrylic-based double-sided pressure-sensitive adhesive (Adhesives Research, 90445Q). The bottom-side liner (*i.e.*, without the kiss-cut) was peeled off to expose the bottom-side adhesive, and then it was pressure bonded by hand to a new Petri dish. Batches of dish/adhesive were placed in a vacuum desiccator overnight to remove any air bubbles that occurred during bonding. These were stored on a shelf until further use. The acrylic (Modulor, Germany) two-part lid included a thin 0.5 mm base that only has fluidic vias and a thicker 8.0 mm reservoir-defining layer. These two parts were solvent bonded together using dichloromethane (Modulor, Germany).

### 5.3 Electrode fabrication

The fabrication of laser-induced graphene (LIG) electrodes coated with pure PEDOT:PSS hydrogels was recently established by our group.^38^ In short, a CO_2_ laser (same as above) was used to carbonize a polyimide sheet (Kapton HN, 75 μm thick) with a rasterization protocol at 4.8 W and 15.2 mm s^−1^. The freshly carbonized LIG was air plasma-treated (Femto, Diener Electronics) for 5 min at 100 W and 10 sccm to make it more hydrophilic and functionalizable. It was then soaked in 10% w/v hexamethylenediamine (HMDA) for 4 h at room temperature. After washing with DI water and drying with nitrogen, it was dip-coated (Nadetech ND-DC Dip Coater) in a 1% w/v hydrophilic polyurethane solution in 90% ethanol (AdvanSource, HydroMed D3) with a retraction speed of 100 mm min^−1^, and then subsequently placed on a hot plate for 1 h. Electrode connection lines (*i.e.*, between the electrical bump pad and electroactive area) were coated with an acrylate-based varnish (Essence 2 in 1, Cosnova). A PEDOT:PSS dispersion (1.3%) with 15% dimethyl sulfoxide was drop-cast (200 μl onto the 12 mm diameter LIG electrodes after being placed onto a hot plate (60 °C overnight, and then 130 °C for 90 min). The electrodes were stored in 1× PBS (Sigma Aldrich, P3813). Electrical connections were done with a pogo-pin assembly (Mill-Max, 858-22-002-30-001101) fitted with M3 threaded inserts. Custom polyethylene terephthalate glycol (PET-G) 3D-printed (Prusa Research, i3 MK3S) parts were made for compression clamping of the pogo pin assembly (seen in green in Fig. 1) and for consistent angled alignment of electrodes into reservoirs (seen in orange in Fig. 1).

### 5.4 Coating of pH-sensitive polymer

Polyaniline (PANI) is polymerized *via* chemical oxidation^72^ by mixing an equal volume and molarity of pre-chilled aniline monomer (1.42 M) in hydrochloric acid (HCl, (1.42 M)) with pre-chilled ammonium persulfate (APS, (1.42 M)) in deionized water and depositing this polymerizing mixture onto the open microchannels (Video S1†). Note that the aniline solution and the APS solution were prepared fresh and were stored at −20 °C for 30 min before mixing to slow down the polymerization in order to allow sufficient time for deposition. The mixture turned from a colorless solution into a deep black hydrogel over the next 30 min (Video S1†). The *in situ* polymerization embeds itself into the Petri dish polystyrene, such that the unbound PANI hydrogel can be washed away leaving behind a thin layer of green PANI (*i.e.*, emeraldine salt).

For calibration, PANI was coated onto small polystyrene Petri dishes (35 mm diameter, Falcon TC-treated). Stock pH buffers ranging from pH 2.69 to 11.25 were made using different combinations of spiking hydrochloric acid (HCl) or sodium hydroxide (NaOH) into primary salt solutions made of either potassium hydrogen phthalate (KHP), potassium dihydrogen phosphate (KH_2_PO_4_), sodium tetraborate (Na_2_B_4_O_7_), or sodium bicarbonate (NaHCO_3_). A benchtop pH meter was used to verify all pH values (VWR pHenomenal pH 1100 L). After depositing 1 mL of the pH buffer into separate PANI-coated dishes, they were imaged on an incubated inverted microscope (Zeiss Axio Observer) with a 20× objective at 37 °C. Images were processed through a Python script to report a hue value. Specifically, the script averages RGB values across the image and reports a corresponding hue. Based on the maximum and minimum values of RGB, the hue is then calculated by using the Python module “colorsys” to change from RGB to HSV coordinates. These hue values were correlated to the known pH values to generate a calibration curve. The curve was fitted with a bi-dose response sigmoidal curve (Origin 2021).

For coating PANI in the DC stimulation device, the polymerization and casting processes are the same, but now performed on the polystyrene-exposed parts of the microfluidic-defining adhesive (Video S1†). After washing away unbound PANI, the liner was then removed and a lid was subsequently added, just like in Fig. 1b. PBS (1×) was degassed in a vacuum desiccator for 30 min to minimize bubble formation in the microchannel. Then, degassed PBS and electrodes were added to the device. Imaging was performed on an incubated microscope over the course of 20-plus straight hours of monophasic DC stimulation (25 μA using a potentiostat/galvanostat (Metrohm, Autolab PGSTAT204)). For the pseudo-converging EF case, a custom-built relay was used between the current source's working electrode lead and the two anodes.

### 5.5 Culturing keratinocytes

Human epidermal keratinocytes immortalized with HPV-16 E6/E7 were acquired courtesy of Prof. Dr. rer. nat. Thorsten Steinberg (Department of Dental, Oral and Jaw Medicine; University Clinics of Freiburg). Keratinocytes were cultured throughout experiments in serum-free keratinocyte growth medium (KGM2, PromoCell, #C-39016) supplemented with a cocktail of factors and CaCl_2_ provided by the same manufacturer (SupplementMix, PromoCell, #C-20011), as well as neomycin (Sigma-Aldrich, #N1142) at a final concentration of 20 μg mL^−1^ and kanamycin (Sigma-Aldrich, #K0254) at a final concentration of 100 μg mL^−1^. The cell culture was incubated at 37 °C and 5% CO_2_ at 95% humidity and routinely passaged when 80% to 90% confluency was reached. The growth medium was exchanged three times per week. For the experiments, keratinocytes were used from passages 34 to 49.

### 5.6 Treatments of keratinocytes to mimic the diabetic phenotype

For experiments with elevated glucose concentrations, a 1 M aqueous stock solution of d (+)-glucose (Sigma Aldrich, #G7021) was added to the culture medium to achieve a desired concentration (6, 12, 25, 50, or 100 mM). The glucose-rich medium was prepared fresh each time before treatment. Confluent cell layers were treated for 24 h before proceeding to the wounding.^48^

For experiments mimicking a diabetic wound environment, a 25 mM stock solution of p38-MAPK inhibitor (Cell Signalling *via* Selleck Chem, Adezmapimod – SB203580, #S1076) in DMSO was added to the culture medium to achieve a desired concentration (0.5, 5, 25, or 50 μM). The inhibitor-containing medium was prepared fresh the same day as treatment. A control condition to test the effect of DMSO on cell viability and migration was established by using the same final DMSO concentration (0.1% v/v) as above, but without an inhibitor. Confluent cell layers were treated for 3 h before proceeding to the wounding.^54^

In order to assess the viability of cells after treatment, live/dead cell double staining was performed with SYTO 16 (Invitrogen, #S7578) and propidium iodide (Invitrogen, #P1304MP). For staining, culture medium containing both dyes with a final concentration of 500 nM each was prepared. Cells were protected from light and incubated at 37 °C for 30 min, then washed with 37 °C PBS and imaged with an incubated inverted microscope (Zeiss Axio Observer).

### 5.7 Seeding cells onto devices, wounding monolayers, and device assembly

Before seeding on the bioelectronic wound healing assay devices, the devices were air plasma-treated (30 W, 3 min, 10sccm) to improve cell adhesion to the substrate. In preparation, keratinocytes were detached from culture flasks by incubation with 0.05% trypsin and 0.02% EDTA solution (Sigma Alrich, #T3924) at 37 °C for 5 min. To neutralize trypsin, medium containing 10% fetal bovine serum (Sigma Aldrich, #F0804) was used. Harvested cells were centrifuged (1200RPM for 10 min) and resuspended at 4.5 × 10^6^ cells per mL^−1^. Cell suspensions (100 μl) were spotted directly over open microchannels (see Fig. 1b, step 3) and incubated for 3 h to allow for cell attachment. Afterwards, the excess of cells was washed away with 37 °C PBS solution and aspirated before adding 10 mL of fresh growth medium. The cell seeding concentration was titrated in order to find the optimal amount so that the devices were fully confluent the next day (Fig. S1†).

Monolayers were scratched using a sterile p10 pipette tip (≃700 μm) that was connected to a vacuum aspirator (Vacusip, Integra Biosciences). The motion of the scratch was always done starting at the base of the microchannel scratch alley and finishing where the four channels converge. After the wound formation, the medium was aspirated, and then the devices were washed with sterile PBS (1×), fresh growth medium was added, and they were finally placed back in the incubator for 4 h. In the meantime, the acrylic lid, electrodes, 3D printed adapters and wires were washed with 70% ethanol and then further sterilized in an S1 cell culture hood (Safe 2020, Thermo Scientific) *via* UV-treatment for 1 h. After incubation, the medium was aspirated until only a small amount of medium resided in the microchannels, leaving the liner as dry as possible in order to minimize the probability of the fluid transferring onto the dry adhesive. The liner was then peeled, the two-part acrylic lid was aligned and fixed using alignment marks, and the medium immediately replenished by initially flowing 100 μl directly into the microchannels to displace trapped air. The rest of the reservoirs were filled using a standard serological pipette. The electrodes were assembled and placed into the reservoir, and the corresponding wires were routed through the lid, which was applied to prevent evaporation.

### 5.8 Imaging and direct current stimulation

Seeded and assembled devices were placed on an incubated inverted microscope (Zeiss Axio Observer with a Definite Focus 2) and maintained at 37 °C and 5% CO_2_. Phase-contrast images were acquired every 10 min using a 5× objective in order to capture the entire microfluidic network. The DC stimulation was carried out in the exact same way as described in the preliminary pH-monitoring experiments (*e.g.*, constant current using a potentiostat/galvanostat – Autolab PGSTAT204).

### 5.9 Statistical analysis

All cell-based experiments were completed in triplicate. All data plots were assembled using the data analysis software Origin 2021, where the shaded regions of the line plots represent the standard deviation. Output images were put through an ImageJ plugin in order to quantify the wound area closure over time.^73^ Kymographs were collected using the ImageJ plug-in KymographBuilder. Cell tracking was done using CellTracker.^74^

## Author contributions

S. S. and M. A. conceived the project. S. S. designed and fabricated all platforms (fluidic and electrochemical). S. S. performed all FEA simulations/analyses and electrode characterization. L. M. and J. L. performed image analysis of PANI-based experiments. A. S., A. K., and N. J. performed all cell culturing, cell seeding, and cell motility inhibition assays. S. S. performed all stimulation-based experiments. S. S. analyzed, compiled, and plotted the data. S. S. made the figures. S. S. wrote the manuscript. All authors reviewed the manuscript.

## Conflicts of interest

There are no conflicts to declare.

## Supplementary Material

LC-023-D2LC01045C-s001

LC-023-D2LC01045C-s002
