# Supplementary material for: Bioelectronic microfluidic wound healing: a platform for investigating direct current stimulation of injured cell collectives
Source: Lab Chip. 2023 Jan 18;23(6):1531–46. doi: 10.1039/d2lc01045c (PMC10013350; doi:10.1039/d2lc01045c)
Supplement: LC-023-D2LC01045C-s002 [file LC-023-D2LC01045C-s002.pdf]

Electronic Supplementary Material (ESI) for Lab on a Chip.
This journal is © The Royal Society of Chemistry 2023

Supplementary Information

Bioelectronic microfluidic wound healing: a platform for investigating direct current stimulation of injured cell collectives

Sebastian Shaner[1,2], Anna Savelyeva[1,2], Anja Kvartuh[1], Nicole Jedrusik[1,2], Lukas Matter[1], José Leal[1,2], Maria Asplund[1,2,3,4,5]

[1]Department of Microsystems Engineering, University of Freiburg, Freiburg, Germany

[2]BrainLinks-BrainTools Center, University of Freiburg, Freiburg, Germany

[3]Freiburg Institute for Advanced Studies (FRIAS), University of Freiburg, Freiburg, Germany

[4]Division of Nursing and Medical Technology, Luleå University of Technology, Luleå, Sweden

[5]Department of Microtechnology and Nanoscience, Chalmers University of Technology, Gothenburg, Sweden

Declarations of interest: none

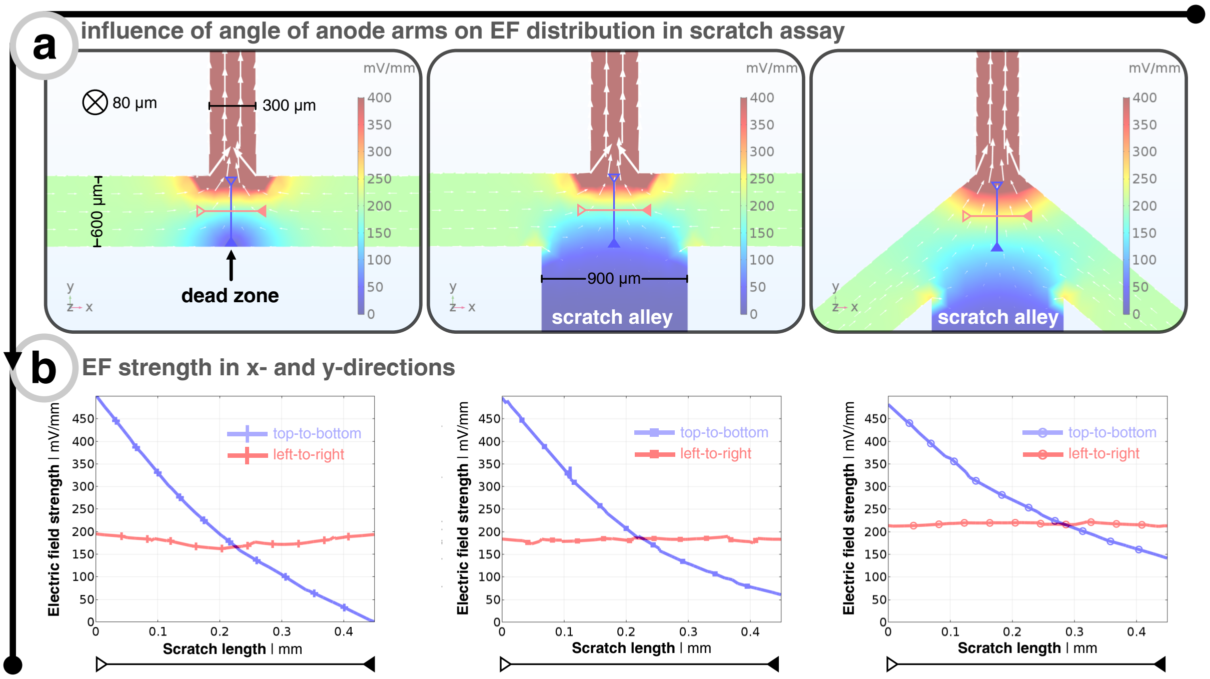

Fig. S1: Finite element analysis reveals the effect of the angle of converging anode arms and scratch alley on EF distribution. (a) Three different configurations of the angle of the left and right microfluidic branches in relation to the top cathode branch and the existence of a scratch alley or not. Note that for all cases, the channel dimensions are consistent between models, there are two shorted anodes (one on both sides of the converging zone), and a single cathode connected to the top channel. (b) Electric field strength of blue and red line sections from (a). The blue line starts from entry of top channel and points downward. The red line starts left of the theoretical scratch zone and ends on the right of said zone. Forcing converging current into the top channel of a T-junction creates a dead zone, seen in left column and blue trace. Adding a scratch alley (middle column) helps minimize the dead zone effect seen in blue trace. The angled anode branches helps even further.

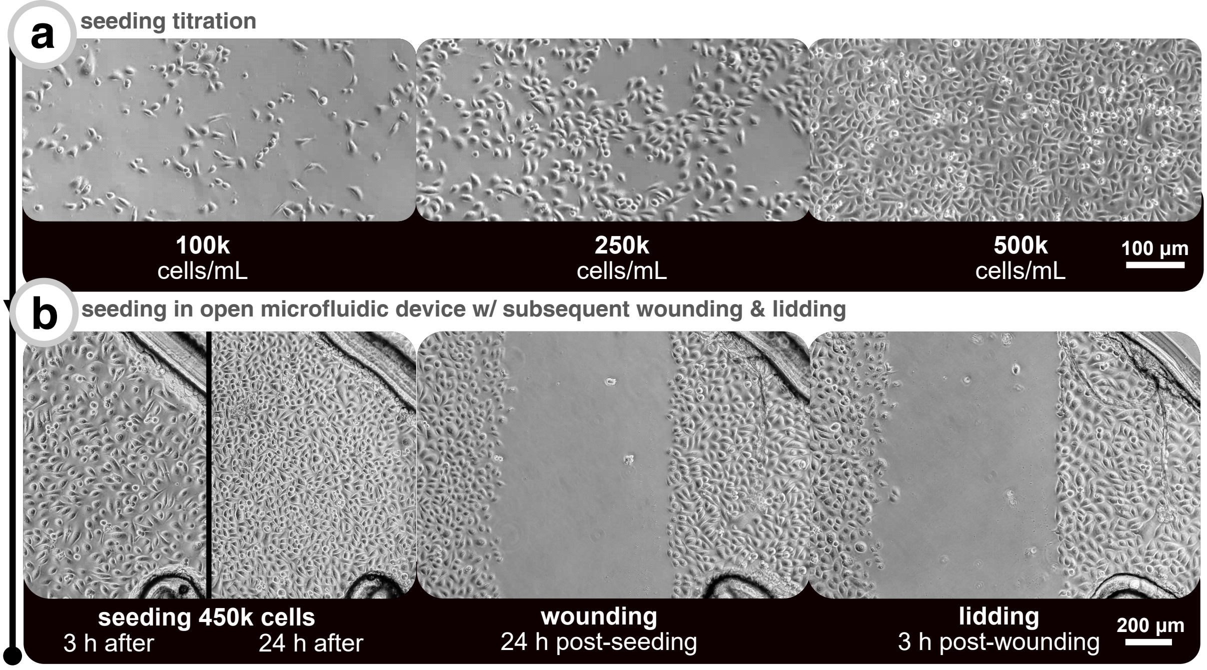

Fig. S2: Seeding titration of keratinocytes to find full confluency. (a) Seeding titration of cell suspension density to find out spatial adherence density 3 hr after spotting cell suspension on substrate. Reminder that the goal was to have a fully confluent layer of cells 24 hr later for scratch experiments. (b) Final cell suspension density (4.5×10^6 cells mL^{-1} in 100 μL) selected for all scratch assay device experiments. First two images show keratinocytes 3 hr and 24 hr after seeding. Next images shows a typical wound directly after scratching and after microfluidic lidding.

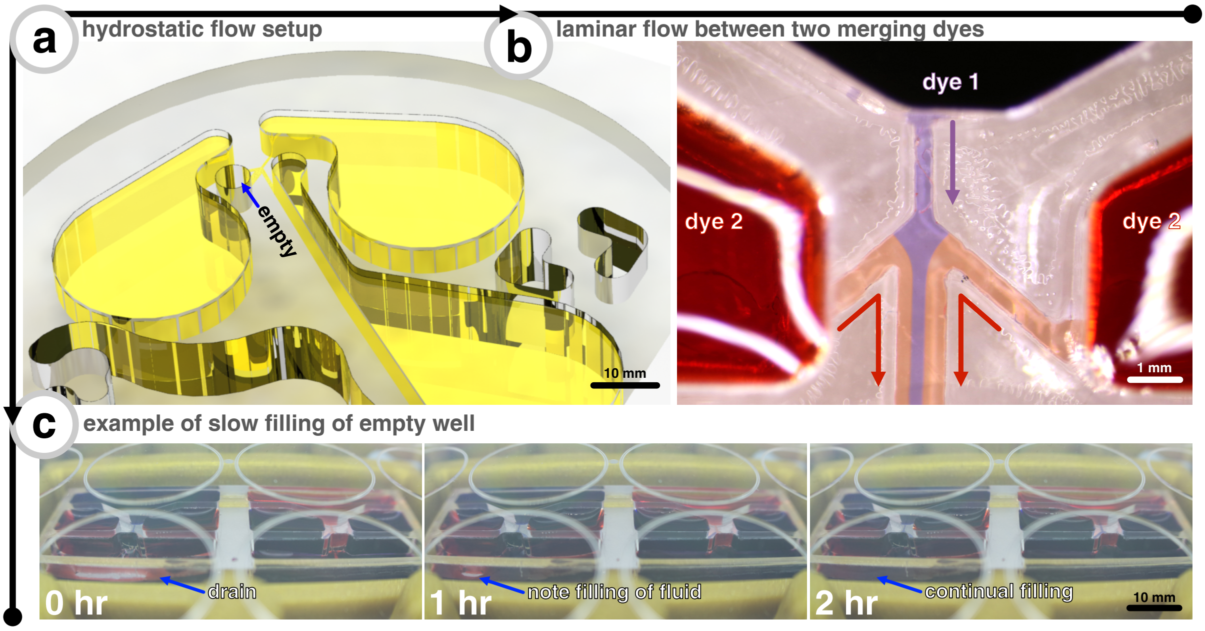

Fig. S3: Hydrostatic flow option in microfluidic scratch assay device. (a) setup of how hydrostatic pressure can be used for passive perfusive flow where the three electrode wells are full and the fourth drain well is empty. (b) adding dye 1 (Brilliant Black BN) in top well and dye 2 (Congo Red) into left and right wells show how flow is laminar when fluids used have different properties. (c) time-lapse example of how fourth drain well slowly fills due to hydrostatic flow.

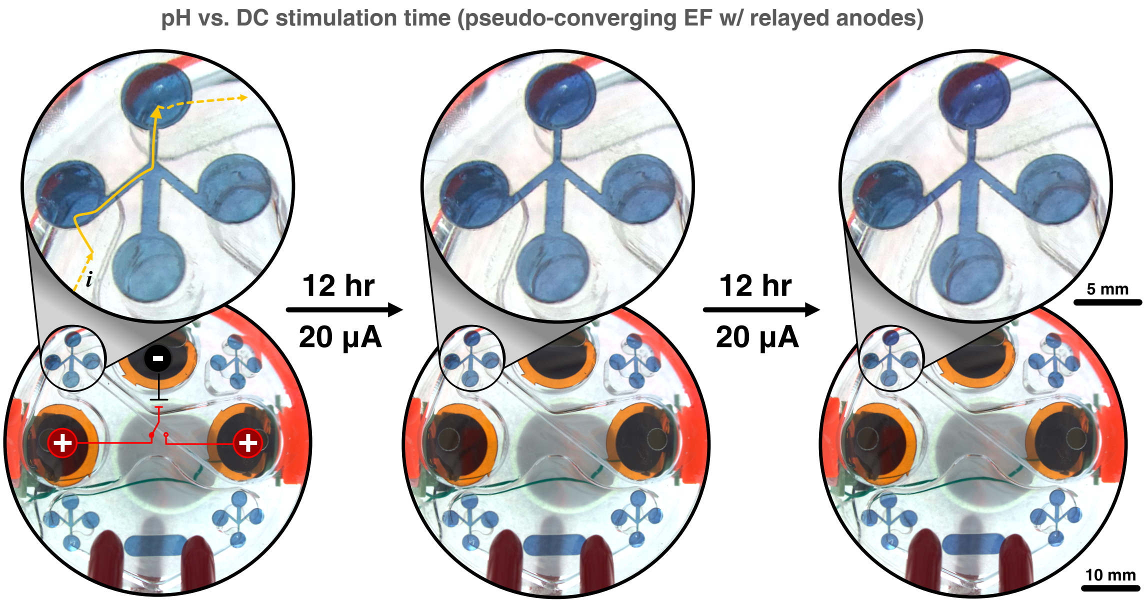

Fig. S4: pH versus DC stimulation time for pseudo-converging EF. Note that stimulation protocol is identical to that of Fig. 3c (main text). The relayed nature of switching between left and right anodes prevent extended time in faradaic current regime, which allows for minimal anode pH shifts (i.e., no noticeable lowering of pH at anodes) for the entire 24 hr of DC stimulation. However, since the cathode is not switched, the cathode well gets more basic (i.e., purple).

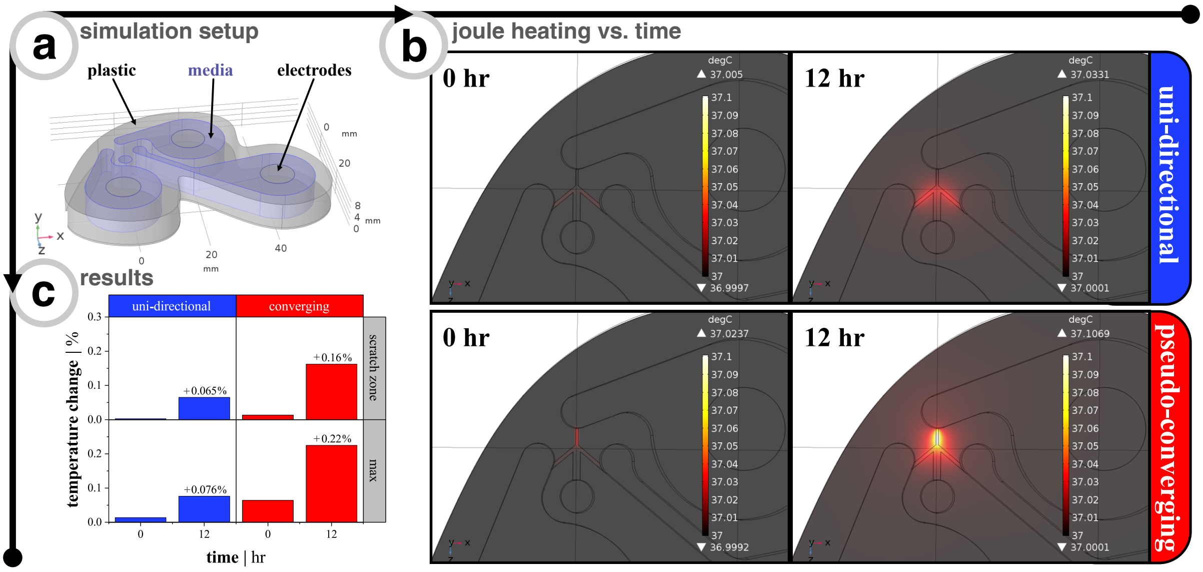

Fig. S5: Simulations of joule heating due to direct current (DC) stimulation. (a) Setup of simulation where the blue shows the electrolyte filled reservoirs surrounded by plastic (i.e., acrylic) and the electrodes sitting on top of the electrolyte. Microchannels that connect the reservoirs are not known here, but can be inferred from Fig. 2 and Fig. 3 (both from the main text). The surrounding temperature boundary condition was set to 37 °C, which serves as the reference point for percent change of temperature. (b) Snapshots of joule heating expected in microchannel for both electrode configurations after 12 hr of constant current stimulation. (c) Simulation results showing minimal joule heating of electrolyte even after long-term stimulation. Whether in the small channels or scratch zone, the heating generated is within 1/10 of a degree Celsius, which is negligible.

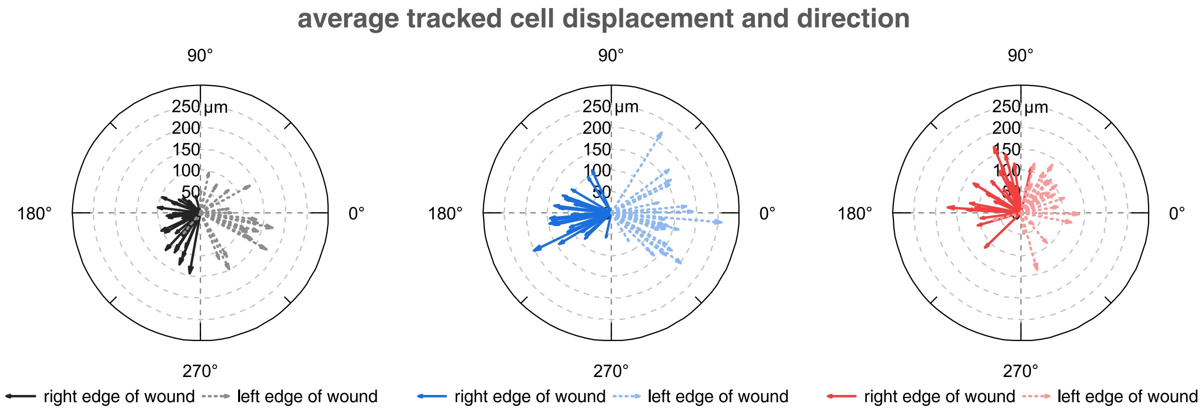

Fig. S6: Cell tracking of keratinocytes with and without DC stimulation. Plot of the average displacement and direction for tracked cells. For the uni-directional EF case, the cathode and anode are at the 0° and 180° locations, respectively. For the pseudo-converging EF case, the cathode is at the 90° location, with the anode switching between the 0° and 180° locations every 30 min. The data coincides with Figure 3 from the main text.

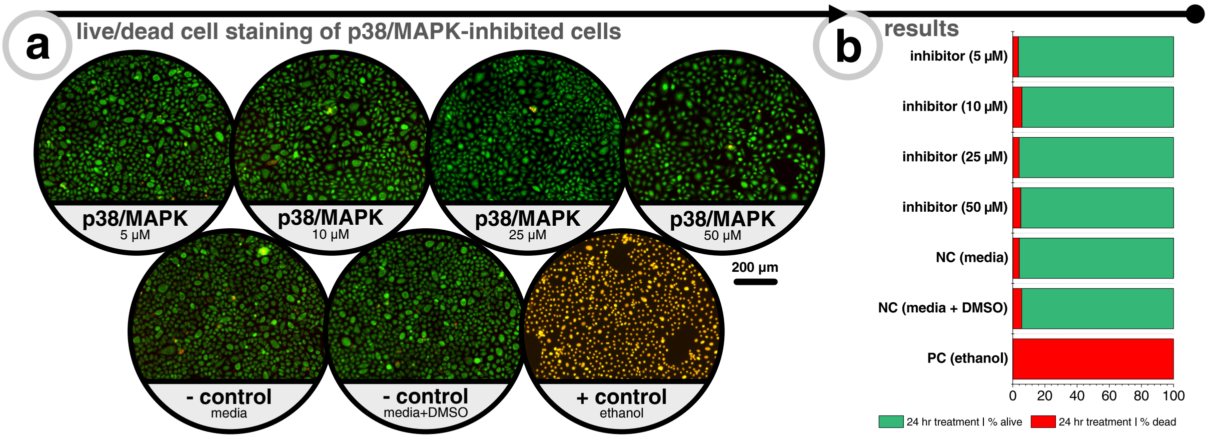

Fig. S7: p38/MAPK-inhibitor treatment does not impact cell viability. (a) Example images of live/dead (SYTO 16 & propidium iodide) cell staining of keratinocytes after 24 hr of treatment. Negative controls were either media or media plus DMSO (with same concentration as inhibitor samples). Positive control was a treatment with 70% ethanol. (b) Results of live/dead staining where green and red signifies percentage of total population that is live and dead, respectively.
